# Immunohistochemical Analysis of Placental Tissue of Women Infected with SARS-CoV-2 During Pregnancy—A Prospective Clinical Study

**DOI:** 10.3390/ijms26157659

**Published:** 2025-08-07

**Authors:** Marija Bicanin Ilic, Tamara Nikolic Turnic, Aleksandar Nikolov, Srdjan Mujkovic, Ivana Likic Ladjevic, Igor Ilic, Marija Spasojevic, Nikola Jovic, Jovana Joksimovic Jovic, Dejana Rakic, Begzudin Ahmetovic, Sara Rosic, Aleksandra Dimitrijevic

**Affiliations:** 1 Department of Gynecology and Obstetrics, Faculty of Medical Sciences, University of Kragujevac, 34000 Kragujevac, Serbia; aleksandar.nikolov2@gmail.com (A.N.); drsrdjanmujkovic@gmail.com (S.M.); docctorny@gmail.com (N.J.); dejavulovic@gmail.com (D.R.); saskadkg@gmail.com (A.D.); 2 Clinic of Gynecology and Obstetrics, University Clinical Center Kragujevac, 34000 Kragujevac, Serbia; 3 Department of Pharmacy, Faculty of Medical Sciences, University of Kragujevac, 34000 Kragujevac, Serbia; tnikolict@gmail.com; 4 N.A. Semashko Public Health and Healthcare Department, F.F. Erismann Institute of Public Health, I.M. Sechenov First Moscow State Medical University (Sechenov University), 119435 Moscow, Russia; 5 Center of Excellence for Redox Balance Research in Cardiovascular and Metabolic Disorders, 34000 Kragujevac, Serbia; jovana_joksimovic@yahoo.com; 6 Clinic of Gynecology and Obstetrics, University Clinical Center of Serbia, 11000 Belgrade, Serbia; ivanalikicladjevic@gmail.com; 7 Department of Gynecology and Obstetrics, Faculty of Medicine, University of Belgrade, 11000 Belgrade, Serbia; 8 Department of Radiology, University Clinical Center Kragujevac, 34000 Kragujevac, Serbia; ilicigor2909@gmail.com; 9 Department of Pathology, Faculty of Medical Sciences, University of Kragujevac, 34000 Kragujevac, Serbia; spasojevicmarija89@gmail.com; 10 Department of Pathology, University Clinical Center Kragujevac, 34000 Kragujevac, Serbia; 11 Department of Physiology, Faculty of Medical Sciences, University of Kragujevac, 34000 Kragujevac, Serbia; 12 Department of Gynecology and Obstetrics, General Hospital Brcko District, 76100 Brcko, Bosnia and Herzegovina; begzudin@hotmail.com

**Keywords:** spike, CD68+ leucocytes, ACE2 receptor, placental inflammation, SARS-CoV-2

## Abstract

SARS-CoV-2 has an affinity for binding to the human Angiotensin-converting enzyme 2 (ACE2) receptor through cleavage and conformational changes at the S1–S2 boundary and the receptor binding domain of the spike protein, which is also the most variable part of SARS-CoV-2. This study aimed to investigate the expression of Angiotensin-converting enzyme 2 (ACE2), spike protein, and CD68+ markers in placental tissue to demonstrate a possible correlation with the level of systemic oxidative stress biomarkers in patients who were infected with SARS-CoV-2 during pregnancy. A prospective clinical cohort study was designed to investigate the presence of CD68+ macrophages, ACE2, and spike proteins in placental tissue using immunohistochemical methods and to compare these results with oxidative stress from our previous study. Spike and CD68+ macrophages’ immunoreactivity were more pronounced in the placental tissue of patients from the SARS-CoV-2 group. Placental tissue spike protein and CD68+ immunoreactivity correlate with maternal and fetal Thiobarbituric Acid Reactive (TBARS) levels. This study has confirmed that spike protein expression in placental tissue is associated with the newborn’s stay in intensive neonatal care. Therefore, immunoreactivity analysis for the Spike antigen is important in detecting newborns at risk of early neonatal complications.

## 1. Introduction

During the COVID-19 pandemic, scientists focused on understanding the pathogenesis of SARS-CoV-2 infection during pregnancy and the potential routes of fetal/neonatal infection. Numerous studies have revealed parts of this complex pathogenesis, but the results have often been conflicting due to inconsistencies in terminology, numerous confusing factors, and potential biases. Still, scientific attention is focused on finding non-expensive, readily available tests that are acceptable for patients and can be performed in a short time to provide us with information on possible maternal or fetal/neonatal risks of COVID-19 complications.

Together with MERS and SARS-CoV, it is one of seven human-infecting viruses from the family of beta-type Coronaviridae [1,2]. SARS-CoV-2 is constituted of an enveloped RNA virus and four structural proteins [3]. Each of these proteins, namely the spike (S) protein, nucleocapsid (N) protein, envelope (E) protein, and membrane (M) protein play distinct roles in pathogenesis [4,5].

SARS-CoV-2 has an affinity for binding to the human Angiotensin-converting enzyme 2 (ACE2) receptor through cleavage and conformational changes at the S1–S2 boundary and the receptor binding domain of the spike protein, which is also the most variable part of SARS-CoV-2 [6]. When the virus encounters a potential host, it initially binds to ACE2 receptors with the help of the spike protein, and the membranes of the virus and the host cell fuse [7,8,9]. These receptors are found on the kidney, endothelium, heart, macrophage, and pneumocyte cells, but are predominant on type 2 alveolar epithelial cells, making them highly sensitive to the virus’s effects [10,11,12]. In addition to the ACE2 receptor, Type 2 Transmembrane Serine Protease (TMPRSS2) also plays a key role. This protease leads to the cleavage of the spike protein as well as a change in shape that facilitates the fusion of the virus’s and the host’s membranes, allowing the virus’s RNA to enter the host cell and initiate replication [13]. The ACE2 receptor, crucial for regulating blood pressure, is also subject to changes during pregnancy. Numerous studies have shown an increased expression of ACE2 during pregnancy, especially in patients who consume tobacco, which affects the increased susceptibility of pregnant women to SARS-CoV-2 infection. An increased number of ACE2 receptors is detected in the tissues of the kidneys, placenta, and uterus [14,15,16,17].

The pathophysiological route of infection on the fetus can be direct, transmitted through the placenta, or indirect, creating unfavorable conditions for the development of the fetus because of inflammation, microthrombosis, and hypercoagulation. Based on these pathophysiological findings, we have investigated the influence of SARS-CoV-2 on placental tissue and oxidative stress in mothers and neonates in our latest study [5]. Numerous studies have also examined macroscopic and microscopic pathohistological changes within the placental tissue in patients infected during pregnancy. Still, the results are incoherent due to equivocal terminology, classification of placenta lesions, and potential researcher biases [18,19,20,21,22,23,24]. Fetal vascular malperfusion (FVM) was the most frequent finding in pregnant patients infected with SARS-CoV-2 [25,26]. In contrast, others have reported maternal vascular malperfusion (MVM) or inflammatory changes [25,26,27,28,29,30]. Placental tissue is rich in both maternal and fetal macrophages, which play an essential role during placentation, the immune response in pregnancy, and the initiation of the delivery mechanism [31,32]. Maternal macrophages express ACE2 receptors on their surface, potentially enabling binding of the SARS-CoV-2 virus and direct transmission to the fetus via the placenta during inflammation, especially during chorioamnionitis [33,34]. Hofbauer cells, which represent placental macrophages, are in the stroma of chorionic villi, localized near fetal vessels and trophoblasts, and possess classic macrophage/monocyte markers such as CD68+ [35,36]. In addition to Hofbauer’s cells, histopathological studies of the placentas of mothers infected with SARS-CoV-2 show that histiocytes are expressed at the decidual level [37]. Histiocytic infiltration of the intervillous space is a characteristic feature of chronic histiocytic intervillitis, a rare pathological entity of unknown etiology often associated with poor perinatal outcomes [38]. Chronic histiocytic intervillitis is characterized by maternal CD68+ cells infiltrating at least 5% of the intervillous space without other signs of infiltration, and the perinatal outcome is correlated with the degree of infiltration [35,39,40,41].

## 2. Results

### 2.1. Clinical Characteristics of Patients with COVID-19 Compared with the Control Group

This study included 50 pregnant women, 28 of whom were SARS-CoV-2 positive (COVID-19 group), and 22 in the SARS-CoV-2 negative (control) group. The average age of the participants was 30.61 ± 4.72 and 31.41 ± 4.65 in the COVID-19 and control groups, respectively. Of the total number of participants, 17.86% of pregnant women were infected during the first trimester, 42.86% during the second trimester, and 39.29% during the third trimester. The mean length of pregnancy was 39.19 ± 0.98 gestational weeks (gws) in the COVID-19 group and 39.42 ± 1.26 gws in the control group.

Subgroups were formed based on the severity of their symptoms; patients with mild COVID-19 were asymptomatic or experienced a cough, sore throat, fatigue, headache, loss of taste or smell, or low-grade fever. Patients with severe COVID-19 had a fever, tachypnea, respiratory distress symptoms, and lung infiltrates during pregnancy. A total of 41.38% of patients experienced mild symptoms, while 58.62% had severe symptoms of COVID-19. Within a group of patients with severe symptoms of COVID-19, three patients developed pneumonia and were dependent on oxygen therapy.

The mean length of pregnancy was similar in both groups: 39.19 ± 0.98 gws in the COVID-19 group and 39.42 ± 1.26 gws in the control group. Delivery was completed vaginally in 69% of cases and by cesarean section in 31% of cases within the SARS-CoV-2+ group, compared with 45% of vaginally completed deliveries and 55% of cesarean sections in the control group.

Statistically, there was no significant difference in the number of positively tested newborns between the groups (*p* = 0.37), in the manner of completion of labor (*p* = 0.29), in the sex of the newborn (*p* = 0.13), as well as in the meconial membranes and meconial amniotic fluid between the groups (*p* = 0.21 and *p* = 0.37).

### 2.2. Immunohistochemical Analysis of the Expression of ACE2 Within the Placental Tissue of Patients Infected with SARS-CoV-2 During Pregnancy

**Figure 1:**
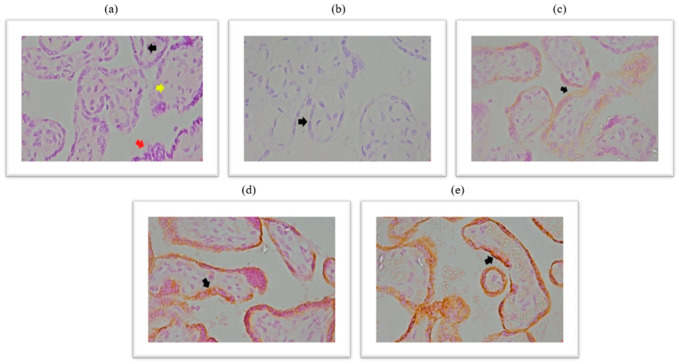
(**a**) Chorionic villi with edematous stroma and dilatated blood vessels, HE stained, magnification 400×; (**b**) trophoblast cells do not show membrane and cytoplasmic immunopositivity on ACE2 antibody-negative control, magnification 400×; (**c**) trophoblast cells show membrane and cytoplasmic immunopositivity on ACE2 antibody of light intensity (light yellow color), magnification 400×; (**d**) trophoblast cells show membrane and cytoplasmic immunopositivity on ACE2 antibody of moderate intensity (light brown color), magnification 400×; (**e**) trophoblast cells show membrane and cytoplasmic immunopositivity on ACE2 antibody of pronounced intensity (dark brown color), magnification of 400×. Black arrow represents dilated blood vessels; yellow arrow represents edematous stroma; red arrow represents cytotrophoblast cells.

Immunohistochemical analysis of placental ACE2 expression is documented in the micrographs shown in Figure 1. HE stained chorionic villi with edematous stroma and dilatated blood vessels (Figure 1a), a negative control micrograph staining without ACE2 antibody (Figure 1b), and with different intensities of ACE2 protein immunopositivity are shown (Figure 1c–e).

**Figure 2:**
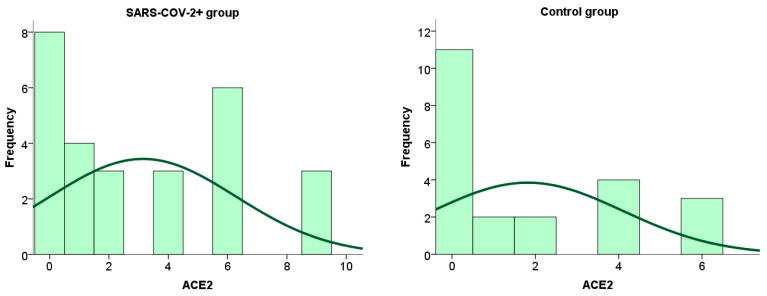
Frequency of ACE2 expression on placental tissue cells in patients infected with SARS-CoV-2 during pregnancy and in the control group.

We performed a statistical analysis of the frequency distribution of the ACE2 immunoreactivity scores in the placental tissue of SARS-CoV-2+ patients and the control group. The results are shown graphically in Figure 2.

### 2.3. Expression of the Spike Protein Within the Placental Tissue of Patients Infected with SARS-CoV-2 During Pregnancy

**Figure 3:**
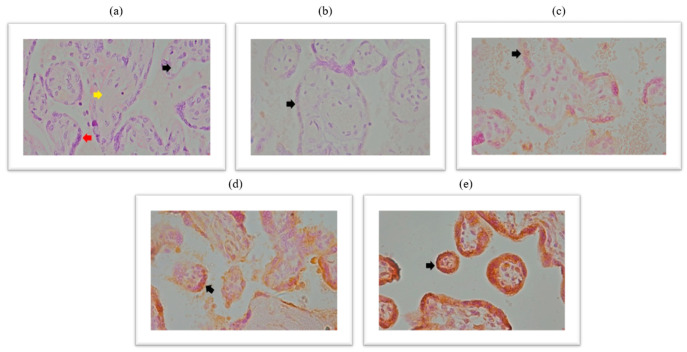
(**a**) Chorionic villi with edematous stroma and dilatated blood vessels, HE stained, magnification 400×; (**b**) trophoblast cells do not show immunoreactivity to SARS-CoV-2 spike antibody-negative control, magnification 400×; (**c**) trophoblast cells exhibit membrane and cytoplasmic immunopositivity of light intensity to SARS-CoV-2 spike antibody (light yellow color), magnification 400×; (**d**) trophoblast cells show membrane and cytoplasmic immunopositivity of moderate intensity to SARS-CoV-2 spike antibody (light brown color), 400× magnification; (**e**) trophoblast cells show membrane and cytoplasmic immunopositivity of pronounced intensity on SARS-CoV-2 spike antibody (dark brown color), magnification 400×. Black arrow represents dilated blood vessels; yellow arrow represents edematous stroma; red arrow represents cytotrophoblast cells.

Immunohistochemical analysis of the placental expression of the spike protein is documented in the micrographs shown in Figure 3. HE stained placental tissue showing chorionic villi with edematous stroma and dilatated blood vessels (Figure 3a) and a negative control micrograph staining without spike antibodies (Figure 3b), together with different intensities of the spike protein immunopositivity are shown (Figure 3c–e).

**Figure 4:**
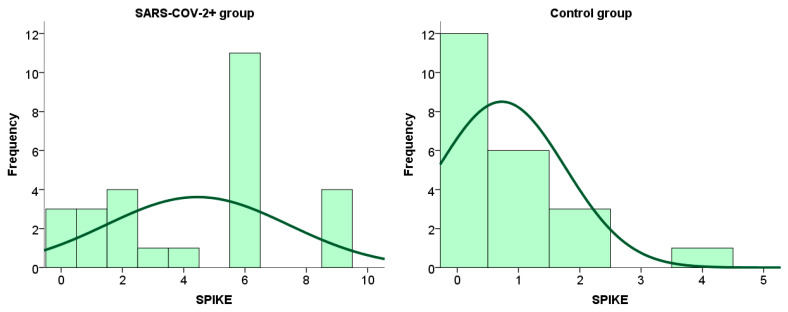
Frequency of spike protein expression within placental tissue in patients infected with SARS-CoV-2 during pregnancy and in the control group.

**Figure 5:**
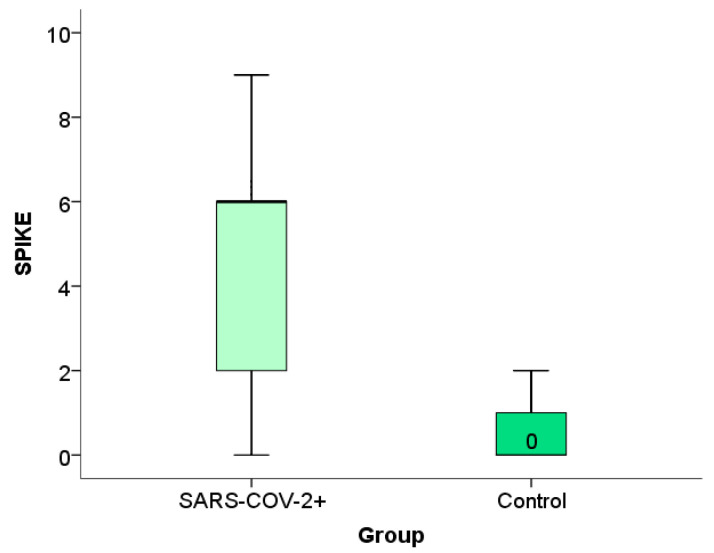
Distribution of frequency of spike protein expression within placental tissue in patients infected with SARS-CoV-2 during pregnancy and in the control group.

We have shown the distribution frequency of spike protein immunoreactivity scores within the placental tissue of SARS-CoV-2+ patients and the control group in Figure 4 and Figure 5.

### 2.4. Expression of CD68+ Macrophages Within the Placental Tissue of Patients Infected with SARS-CoV-2 During Pregnancy

**Figure 6:**
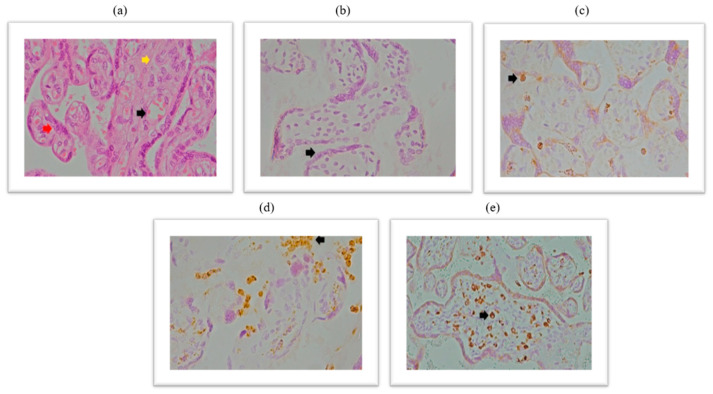
(**a**): The 1a chorionic villi with edematous stroma and dilatated blood vessels, HE stained, magnification 400×; (**b**) negative control, magnification 400×; (**b**) rare CD68+ macrophages in stroma and intervillous spaces, magnification 400×; (**c**) numerous CD68+ macrophages are present in the intervillous spaces, covering more than 5% of the intervillous space-chronic histiocytic intervillitis, magnification 400×; (**d**) chronic villous histiocytosis, magnification 400×. The black arrow represents dilated blood vessels; yellow arrow represents edematous stroma; red arrow represents cytotrophoblast cells. Black arrow (**d**,**e**)—numerous Hoffbauer cells–fetal macrophages located in stroma of villi.

Immunohistochemical analysis of placental CD68+ expression is documented in the micrographs shown in Figure 5. HE stained placental tissue showing chorionic villi with edematous stroma and dilatated blood vessels (Figure 6a), and a negative control micrograph staining without spike antibody (Figure 6b) is shown together with different levels of CD68+ immunopositivity (Figure 6c–e).

**Figure 7:**
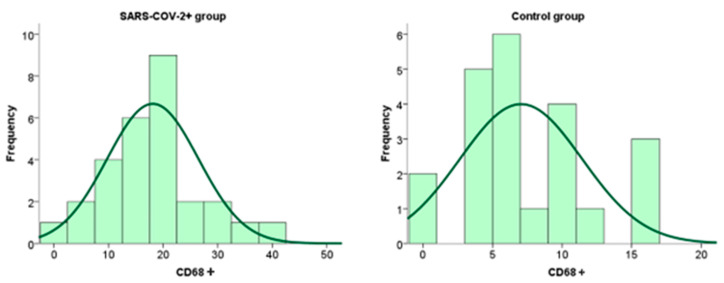
Frequency of expression of CD68+ macrophages within placental tissue in patients infected with SARS-CoV-2 during pregnancy and in the control group.

**Figure 8:**
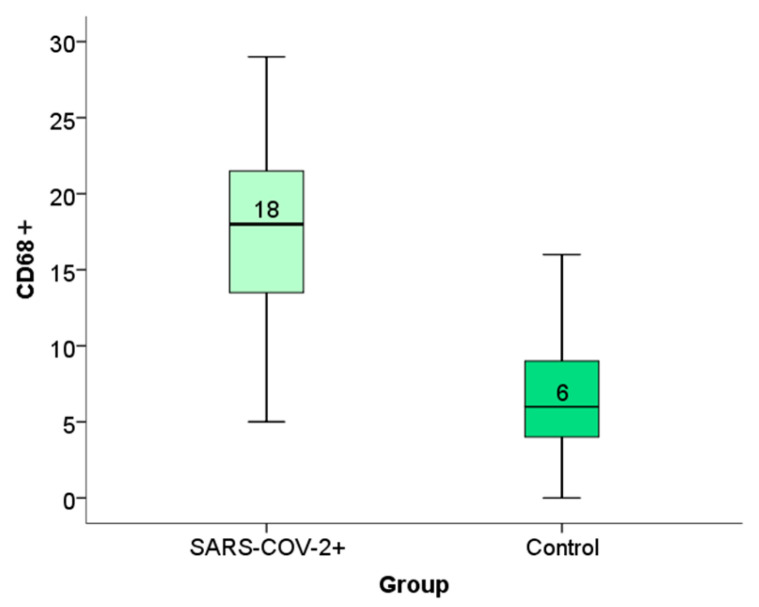
Distribution of the degree of expression of CD68+ macrophage immunoreactivity within placental tissue in patients infected with SARS-CoV-2 during pregnancy and in the control group.

We have shown the distribution frequency of CD68+ immunoreactivity scores within the placental tissue of SARS-CoV-2+ patients and the control group in Figure 7 and Figure 8.

Based on the results from immunopositivity scoring (Table 1), we performed a statistical analysis to compare the expression of spike, ACE2, and CD68+ cells within the placental tissue of patients infected with SARS-CoV-2 during pregnancy with that of the control group.

Based on the results of the Man–Whitney test, we observed a statistically significant difference in the expression of spike proteins and CD68+ macrophages between the SARS-CoV-2+ group and the control group. (*p* < 0.05) (Table 2).

The statistical assay used in this analysis—Chi-square test—showed that there were no significant differences in the expression of ACE2, spike proteins, and CD68+ macrophages within the placental tissue of patients infected with SARS-CoV-2 virus during pregnancy compared with the trimester in which the infection occurred (tested for significance level *p* < 0.05 (Table 3).

The result of the statistical test used in this analysis (Mann–Whitney test) suggests that there are no statistically significant differences in the expression of ACE2 receptors, spike proteins, and CD68+ macrophages within the placental tissue of patients infected with SARS-CoV-2 virus during pregnancy compared with the severity of the maternal clinical picture (for the significance level *p* < 0.05) (Table 3).

### 2.5. Examination of the Association of Perinatal Outcome with the Moment of Disease

For the analysis of categorical variables regarding the gestational week when SARS-CoV-2 infection occurred during pregnancy, the Mann–Whitney U test was employed, and a box plot (rectangular graph) was used to visually represent statistically significant results.

**Figure 9:**
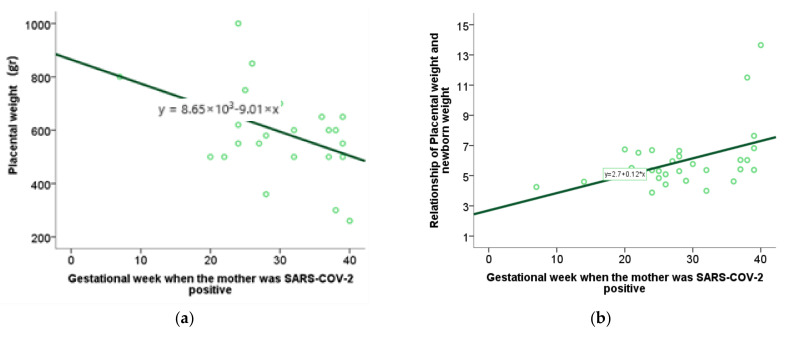
(**a**) Correlation between placental weight and week of gestation in which the infection occurred (*p* = 0.17); (**b**) correlation between the ratio of placental weight to child weight and week of gestation in which the infection occurred (*p* = 0.33).

For the analysis of numerical variables related to the gestational week in which the infection occurred, the method of correlation and regression was applied. Specifically, the value of the Spearman correlation coefficient was interpreted, and a scatterplot was used to graphically represent the statistically significant results. By testing the characteristics of the perinatal outcome using the Spearman range correlation test with the moment at which SARS-CoV-2 infection occurred during pregnancy, we found an association between placental weight and the week of gestation in which the infection occurred, as well as a relationship between placental weight and newborn weight and week of gestation in which the infection occurred (Table 4, Figure 9a,b). Other examined features did not show a statistically significant correlation.

### 2.6. Investigation of the Association Between Spike Protein Expression in the Placental Tissue of Infected Patients and the Level of Maternal and Fetal Oxidative Stress Biomarkers

We have been examining the association between biomarkers of oxidative stress and antioxidant protection in mothers and newborns from our previous study [5] and the expression of the spike protein within placental tissue. A positive correlation was observed between maternal serum TBARS levels and the degree of spike protein’s immunoreactivity within placental tissue. (*p* = 0.04, *r* = 0.29) (Table 5).

### 2.7. Assessment of the Association of Spike Protein and CD68+ Expression with Perinatal Outcome

**Figure 10:**
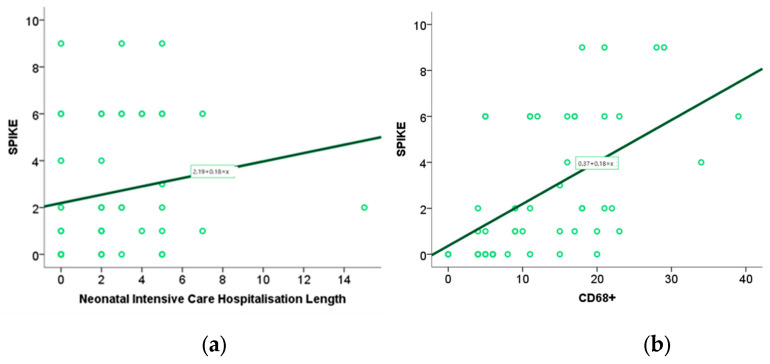
Correlation of the spike protein expression within placental tissue with the stay of the newborn in neonatal intensive care (**a**) and with the expression of CD68+ macrophages (**b**).

**Figure 11:**
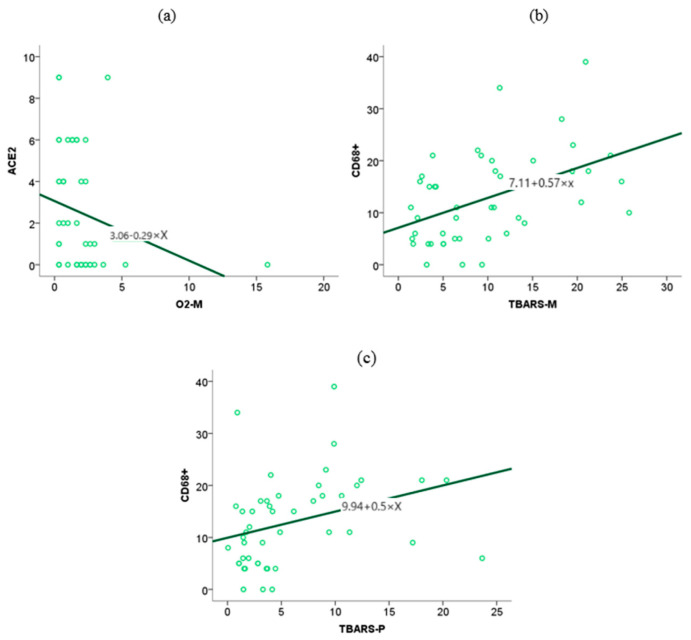
Correlation curve between ACE_2_ with O_2_^−^ anion expression in maternal serum (**a**), CD68+ macrophage expression in placental tissue with maternal serum TBARS levels (**b**), CD68+ macrophage expression in placental tissue with umbilical cord blood TBARS level (**c**).

Using Spearman’s rank coefficient test, we examined the association between perinatal outcomes from our previous study and spike expression within placental tissue, with a significance level of *p* < 0.05. The newborns’ stay in intensive care positively correlates with the spike protein’s expression level in placental tissue (Figure 10) (*p* = 0.02, *r* = 0.33)**.** The correlation and regression methods were applied to the analysis of IHH numerical variables scores, i.e., the value of the Spearman correlation coefficient. A scatter diagram was used to visually present statistically significant results. Through the Spearman correlation test, we examined the association between the expression of spike proteins within the placental tissue of patients infected with SARS-CoV-2 and the expression of CD68+ macrophages and the expression of ACE2 receptors within the same tissue. We found a positive correlation between the expression of spike proteins and the expression of CD68+ macrophages (*p* = 0.00, *r* = 0.60) (Figure 11). In contrast, no statistically significant correlation was observed between ACE2 levels and spike protein levels within placental tissue. (*p* = 0.10) (Figure 10a,b)

### 2.8. Analysis of the Association Between ACE2 Receptor Expression Within Placental Tissue and Blood Biomarkers of Oxidative Stress in Mothers and Newborns After SARS-CoV-2

A statistical analysis was performed to investigate the association between ACE2 expressions in placental tissue and biomarkers of oxidative stress in mothers and newborns using the Spearman rank correlation coefficient, with a significance level set at *p* < 0.05.

The level of ACE2 expression in placental tissue is associated with elevated concentrations of O2- anions in maternal blood, whereas other biomarkers of oxidative stress do not exhibit a similar association (Figure 11).

The association between CD68+ macrophage expression in placental tissue and maternal and neonatal oxidative stress biomarkers, as identified in our previous study, was investigated using the Spearman rank correlation test. The test results showed an association between TBARS in maternal (*p* = 0.00, *r* = 0.48) and fetal serum (*p* = 0.00, *r* = 0.44) and the expression of CD68+ in placental tissue. (Figure 11).

### 2.9. Investigation of the Association Between Pathohistological Findings in Placental Tissue of Patients Infected with SARS-CoV-2 and the Level of Maternal and Fetal Oxidative Stress Biomarkers

**Figure 12:**
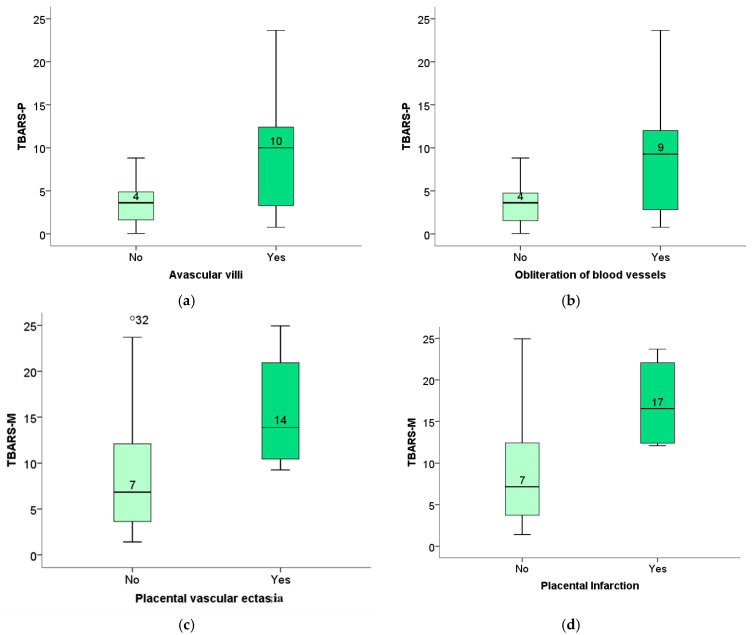

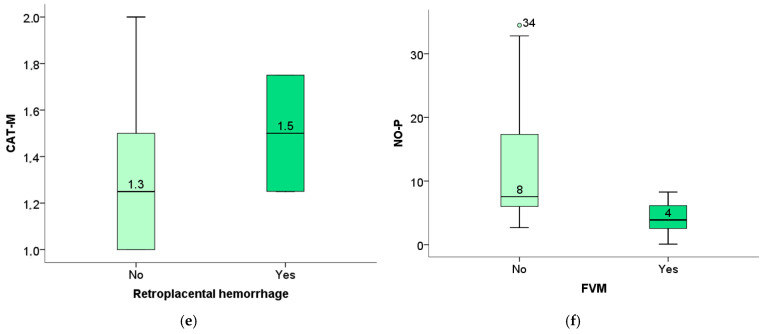
(**a**) The level of TBARS of the newborn concerning the presence of avascular villi (*p* = 0.05); (**b**) the association between TBARS of the newborn and obliteration of blood vessels (*p* = 0.04); (**c**) the association between maternal TBARS and vascular ecstasy (*p* = 0.03); (**d**) the association between maternal TBARS and placental infarction (*p* = 0.03); (**e**) the association between the level of maternal catalase and retroplacental hemorrhage (*p* = 0.03); (**f**) the association between the newborn NO of the fetus with the presence of FVM (*p* = 0.00).

The Mann–Whitney U test was used to analyze oxidative stress parameters in relation to histopathological variables, utilizing data from our previous study [5]. The statistically significant results were compared using a box plot (rectangular graph). We tested the correlation of significant histopathological findings of placental tissue of patients with SARS-CoV-2 with the level of biomarker oxidative stress of mothers and newborns and obtained the following results:

The level of TBARS of the newborn was significantly elevated in pregnant women with placental pathological findings of avascular villi (Figure 12a), as well as obliteration of blood vessels (Figure 12b). In contrast, the level of TBARS of the mother positively correlated with the findings of vascular ecstasy (Figure 12c) as well as placental infarction (Figure 12d).

The level of maternal catalase is positively correlated with the placental findings of retroplacental hemorrhage (Figure 12e), and the level of the fetus’s NO positively correlated with the existence of FVM changes (Figure 12f).

## 3. Discussion

The placenta is an organ that, through its protective mechanisms, prevents the propagation of the SARS-CoV-2 virus to the fetus. However, inflammatory or vascular lesions can weaken the placental barrier, thereby favoring direct transmission [42]. There are several potential maternal–fetal routes of spreading infection. A fetal infection can occur within the uterus by direct transplacental transmission. Another possible way is an intrapartum infection (contact of the newborn with infected vaginal secretion or blood), and the third way is after birth through direct contact between mother and newborn or by breastfeeding [43,44]. Although placental lesions are not specific to SARS-CoV-2 infection, they can be a valuable clue in identifying patients who may develop long-term consequences that require special attention [45,46]. IHC analysis of the spike protein, ACE2 receptors, and CD68+ immunoreactivity can provide us with more specific information, which, together with morphological changes, could be essential in discovering a disruption of placental function and the possibility of transplacental transmission [47]. Genetic disorders significantly impact placental changes and fetal/neonatal outcomes, making it more valuable to analyze these parameters if we had exclusion criteria for abnormal genetic results. Cell-free fetal DNA, as a non-invasive prenatal diagnostic tool for genetic disorders, is a valuable information resource and should be considered an inclusion criterion in future studies [48].

Based on the severity of symptoms, 41.38% of patients had mild symptoms, while 58.62% had severe symptoms of COVID-19, of whom three patients were hospitalized for pneumonia treatment. Although elective admissions were suspended, there was no significant decrease in admissions to the obstetric clinic during the period of our study, similarly to Riemma’s study [49]. Studies examining the impact of SARS-CoV-2 infection during pregnancy showed a positive correlation between the severity of COVID-19 symptoms and pronounced lesions in placental tissue, as well as an increase in oxidative stress during pregnancy [50,51]. The results of our study did not show a statistically significant difference in the frequency of pathohistological changes in placental tissue among the subgroups classified according to the severity of the clinical presentation. Our previous study found no statistically significant differences in biometry parameters between newborns from the SARS-CoV-2-positive and control groups; however, oxidative stress biomarkers were significantly increased in mothers and neonates from the SARS-CoV-2 groups [5]. The mean age of participants in the SARS-CoV-2 group was not significantly different from that of the control group. Mothers’ age could potentially influence the oxidative stress level in neonates [52]. This study provided additional information regarding the relationship between oxidative stress biomarkers and placental lesions, as well as spike, ACE2, and CD68+ immunoreactivity and perinatal outcome.

Decreased placental weight may indicate altered metabolic function and a potential risk to the fetus. Our study, consistent with previous studies, found no significant differences in placental weight and the P/TT (placenta-to-fetal-body weight) ratio between subjects in the SARS-CoV-2+ and control groups [53]. Through additional analysis, we determined the association between the week of pregnancy during which the mother was infected and the altered weight of the placenta, as well as the ratio of placental weight to fetal body weight. The earlier the infection occurred in gestation, the lower the placental weight and the ratio of placental weight to fetal body weight, which may indicate a worse perinatal outcome [54,55,56,57]. Our study also found a positive correlation between placental lesions and oxidative stress biomarkers, highlighting the role of oxidative stress in the pathogenesis of placental lesions. Neonatal TBARS correlate with placental findings of avascular villi or occlusion of blood vessels, and maternal TBARS show a positive correlation with placental findings of avascular villi or occlusion of blood vessels, as well as with vascular extravasation and placental infarction. This finding highlights TBARS as a potentially viable biomarker in the prenatal diagnosis of potential perinatal or neonatal complications.

Numerous studies revealed the complex role of ACE2 receptors in the pathogenesis of COVID-19 [9,12,33,34,57,58]. The enzymatic activity of the ACE2 receptor is increased during pregnancy to ensure adequate circulation to the placenta through the regulatory mechanism renin–angiotensin–aldosterone, by reducing peripheral blood pressure by converting angiotensin I and II to angiotensin (1–7) and angiotensin (1–9) [5,17,33,59,60,61]. Some studies have shown dynamic changes in the expression of this receptor throughout gestation in placental and fetal cells, making them susceptible to SARS-CoV-2 virus invasion [54,57]. This susceptibility is attributed to the presence and coregulation of TMPRSS2, which is necessary for direct transmission in addition to the ACE2 receptor [17,57]. Since this receptor is also the entry site of the SARS-CoV-2 virus into the cell, the virus’s binding leads to competitive inhibition of the receptor responsible for vasodilation and activation of the complement system, resulting in endothelial dysfunction and the formation of microthrombi in this prothrombotic environment [33,58,62,63]. ACE2 is not only a receptor responsible for cytokine production, immune response activation, and viral genome replication, but it is also involved in the regulation of two crucial hormonal systems, the RAS (Renin–angiotensin) and the KKS (Kinin–Kallikrein–Kininase) systems, during pregnancy [14]. Decreased function of the ACE2 receptor is associated with the onset of preeclampsia and impaired fetal growth due to inadequate placentation and hemodynamic adaptation to pregnancy [33,64,65,66,67]. Our study did not detect a statistically significant difference in the expression of ACE2 receptors in the placental tissue of patients infected with SARS-CoV-2 during pregnancy and the control group, nor between the subgroups formed according to the severity of the clinical presentation or the trimester in which the infection occurred but confirmed a positive correlation between the level of ACE2 expression in placental tissue and elevated concentration levels of O2-superoxide anion radicals in maternal blood. Our data supports the fact that patients with a pronounced expression of ACE2 receptors are more susceptible to oxidative stress and the potential complications of COVID-19. Heckt et al. found that SARS-CoV-2 does not result in specific macroscopic or histopathological changes in placental tissue and demonstrated the presence of ACE2 receptors on the membranous side of the syncytiotrophoblast of the chorionic villi as well as on the membranous side of the cytotrophoblast and extravillous trophoblast without expression in the placenta [68,69].

Under physiological conditions, the placenta serves as an effective barrier against the direct transmission of the SARS-CoV-2 virus [42]. Numerous studies, including ours, have demonstrated the impact of SARS-CoV-2 on placental tissue and the fetus; however, the direct transmission of the virus remains a subject of scientific controversy. Data from the literature indicate that direct transmission was detected in only 4–8% of cases [44,70,71,72,73,74,75].

Further, our research was focused on the immunohistochemical detection of the spike protein’s presence and localization within placental cells, revealing a statistically significant difference in spike protein expression between pregnant women infected with SARS-CoV-2 and the control group. Immunoreactivity was observed to the highest degree within cytotrophoblast cells, followed by syncytiotrophoblasts and intervillous mononuclear cells. According to experiences from studies dealing with other viral congenital infections, such as the ZIKA virus, we expected that placental lesions and immunoreactivity would be more pronounced in placentas infected during the first trimester [76]. There were no statistically significant differences in the expression of spike within the placental tissue between the groups divided according to the trimester in which the infection occurred and the severity of the clinical presentation. Our study revealed no differences in immunoreactivity for ACE2, spike protein, or CD68+ expression in relation to the timing of infection. In addition, we found a positive correlation between immunoreactivity to the spike protein within placental tissue and maternal serum levels of TBARS, which indicate an elevated level of oxidative stress in mothers with identified placental SARS-CoV-2 infection. TBARS is important in identifying patients at high risk for complications in pregnancy and the neonatal period. Regarding perinatal outcome, the newborn’s stay in neonatal intensive care positively correlates with the spike protein’s expression level in placental tissue. IHC immunoreactivity analysis for spike antigen is significant in detecting newborns at risk of developing perinatal complications.

Still, the literature’s data on the detection of SARS-CoV-2 within placental tissue are inconsistent. Some studies detected the virus in 100% of the studied samples, while others denied its presence within placenta-infected samples [68,77,78]. A study by Taglauer et al. detected the presence of the SARS-CoV-2 spike protein within villi cells and ACE2 in the outer layer of the syncytiotrophoblast [77]. Other studies addressing similar issues have detected the presence of spike proteins within syncytiotrophoblast cells, with significantly lower expression in cytotrophoblasts, stromal cells, endothelial cells, and innervated mononuclear cells [30,79,80]. In their study, Verma and colleagues showed that significantly higher levels of spike protein were detected within the placentas of preterm deliveries, in trophoblasts, and Hofbauer cells [69]. The study conducted by Guo et al. also demonstrated increased immunoreactivity to the spike protein within the endothelial cells of placental samples from infected patients using the IHC method [81]. From the above, it can be concluded that spike protein-induced release of cytokines and chemokines triggers the formation of cytokine storms, a key factor in the development of placental lesions and maternal and fetal complications. In contrast, elevated levels of tissue factor 3 contribute to thrombotic events and vascular placental lesions [81]. Definitive evidence of placental infection is the presence of the active replication of the SARS-CoV-2 virus within the cells of the placenta. Indeed, the focus of future research on transmission must be to demonstrate active replication [72]. In his study, Incognito concluded that the Delta variant was linked with unfavorable maternal and neonatal outcomes and more frequent placental detection of SARS-CoV-2 [82].

Our study showed a statistically significant difference in CD68+ macrophage expression within placental tissue in patients infected with SARS-CoV-2 during pregnancy compared with the control group. No statistically significant differences were detected between the subgroups regarding the clinical presentation or the trimester in which the infection occurred. In addition to Hofbauer cells, our study also observed the presence of immunoreactivity in the intervillous space, indicating histiocytic intervillitis. Earlier immunohistochemical studies demonstrated intense staining with the CD68+ marker at the level of intervillous spaces, indicating the presence of histiocytic intervillitis in patients infected with the SARS-CoV-2 virus, which should be distinguished from the detected Hofbauer cells of fetal origin that are also CD68 positive [83]. Chronic histiocytic intervillous is characterized by maternal CD68+ cells infiltrating at least 5% of the intervillous space without other signs of astonishment, and the perinatal outcome is correlated with the degree of infiltration [35,39,40,41]. Future studies should consider further differentiation of inflammatory cells using immunohistochemical markers to determine the presence of macrophages, monocytes, and neutrophils, as well as their activated states. In addition, we investigated the associations of CD68+ macrophage expression in placental tissue with biomarkers of maternal and neonatal oxidative stress. We have found a positive correlation between TBARS in both maternal and fetal serum and the expression of CD68+ biomarkers in placental tissue. These data lead to the conclusion that immunoreactivity testing for CD68+ macrophages within placental tissue significantly identifies newborns with possible early or delayed complications of oxidative stress and inflammation.

Future studies should focus on the co-expression of these two proteins and their impact on the outcome of pregnancy [55] as well as on variants of the virus, considering differences in prognosis and outcomes. It is also essential to point out that data on vaccination were the exclusion criterion from our study because they were a possible confounding factor. Additionally, vaccination acceptance among pregnant women in Serbia was very low, likely due to a lack of adequate information, which is crucial for acceptance [5,84].

## 4. Materials and Methods

### 4.1. Study Design

The research was designed as a prospective clinical cohort study that focused on 50 pregnant women who gave birth at the Obstetrics Department of the University Clinical Center Kragujevac from West and Central Serbia, aged 18–42, followed from the moment of a positive COVID-19 test to the discharge from the maternity ward and a newborn from the Neonatology Department. The study lasted 24 months, and all analyses were conducted during hospitalization and outpatient examinations.

### 4.2. Sample Characteristics and Inclusion/Exclusion Criteria

This research study involved 50 pregnant women aged 18 to 42, who delivered vaginally (with or without episiotomy) or by cesarean section at the Obstetrics Department of the GOC UCC Kragujevac. The first experimental group consisted of 28 pregnant women who had a symptomatic or asymptomatic form of COVID-19 during their current pregnancy with a proven presence of SARS-CoV-2 through an RT-PCR test or a rapid antigen test. The control group consisted of 22 pregnant women who also delivered vaginally (with or without episiotomy) or via cesarean section and who denied having COVID-19 during pregnancy, denied close contact with an infected person in the last 7 days, and had a negative serological finding of IgM and IgG antibodies to SARS-CoV-2 from blood sampled upon admission to the Obstetrics Department for delivery.

The main inclusion criteria were: signed form on informed consent for participation in the study of pregnant women aged 18 to 42 admitted to the Obstetrics Department of the Clinic of Gynecology and Obstetrics of the University Clinical Center in Kragujevac (CGO UCC) for pregnancy and childbirth control; patients admitted for childbirth to the CGO UCC Kragujevac who had a positive result of RT-PCR and/or a rapid antigen test for SARS-CoV-2 during pregnancy. The main exclusion criteria were: positive personal history of the presence of thromboembolic diseases; thrombophilia, antiphospholipid syndrome, and the use of anticoagulant therapy; patients suffering from diabetes mellitus; systemic lupus erythematosus, hypertensive disorders during pregnancy or pre-existing chronic hypertension; patients whose pregnancy is complicated by RH incompatibility; patients who are active smokers or have consumed cigarettes in the previous 3 years; patients vaccinated before or during pregnancy against COVID-19; violation of research protocol and voluntary abandonment of participation in the study.

### 4.3. Immunohistochemical Analysis

Immunohistochemical analysis was performed using placental tissue to detect the presence of CD68+ macrophages, ACE2, and spike proteins according to the manufacturer’s guidelines. First, we proceeded with the deparaffinization and rehydration of tissue sections, and then we puffed cuvettes with the glass in question using clips in citrate buffer (pH 6.0) at room temperature. After validation, the preparations were rinsed first with distilled water and then with a PBS (Phosphate-Buffered Saline) solution of pH 7.2. They were then treated with acetone and finally rinsed with PBS on three occasions. The next step before incubation was the application of drops of hydrogen peroxide block, followed by rinsing in a PBS solution. Before primary antibody application and incubation, tissue slices were treated with a commercial protein block to prevent the binding of specific non-specific proteins. The primary antibodies we used were: Anti-ACE2 antibody (ab108252), Abcam; Anti-CD68+ antibody (ab213363), Abcam; SARS-CoV-2 spike glycoprotein antibody—Coronavirus 7 (ab272504), Abcam. A secondary antibody was used in the study: Goat Anti-Rabbit IgG H&L (ab6721) (HRP), Abcam [85].

The next step in the preparation was casting excess antibodies and applying a secondary antibody, followed by a 45 min incubation period at room temperature. Further processing with PBS solution was followed by the application of an HRP/AEC reagent solution and examination with distilled water. The final step was Mayer’s hematoxylin staining [86]. After the final staining and rinsing, the preparations were treated with glycerol and covered with a cover slip. They were analyzed independently by two experienced pathologists, who specialized in placental pathology, using a light microscope of the Olympus BX51 brand, Japan, at magnifications of 50×, 100×, and 400×. Photomicrographs were also performed during the analysis. In case of inconsistent results, the position of the third pathologist was decisive.

### 4.4. Clinical Scoring of Immunohistochemical Data

Scoring of immunohistochemical results in the analysis of Anti-ACE2 antibody expression and SARS-CoV-2 spike glycoprotein antibody expression was performed by manually counting the following parameters, as presented in Table 1. Multiplying the intensity score by the percentage representation score yields a combined score, which provides a more accurate interpretation of the results.

Scoring in the expression analysis of Anti-CD68+ antibodies was performed quantitatively by manually counting CD68+ cells in 5 adjacent High-Power Fields at 400× magnification. We calculated the mean value of the sum of CD68+ cells in 5 adjacent fields. For the sake of standardization, we have assigned scores to mean values, as presented in Table 6.

### 4.5. The Oxidative Stress Biomarkers Data

The oxidative stress biomarker data used in this manuscript originated from the last part of our study, which investigated the connection between maternal and fetal redox status after SARS-CoV-2 infection and outcome [5]. The Ethical Board of the University Clinical Center Kragujevac approved the re-use of those data.

### 4.6. Statistical Analysis

In the statistical processing of the data, the parameters of descriptive statistics were used: frequencies, percentages, mean, median, standard deviation (SD), and range. The statistical significance of numerical variables is determined by Student’s T test or the Mann–Whitney test, depending on the type of data distribution. To analyze categorical variables according to a group of patients, a chi-square test for independence was used. To analyze the numerical variables related to the trimester, the Kruskal–Wallis test was employed. Statistical tests (e.g., correlation) were used based on the results of the baseline, exploratory analysis. Using Spearman’s correlation coefficient, the effect of the investigated independent variables on study outcomes was examined. For the analysis of numerical variables related to gestational age, the method of correlation and regression was applied, i.e., the value of Spearman’s correlation coefficient was interpreted, and the statistical significance of the probabilities of the examined differences in variable values was assessed. The difference between study groups was assumed to be statistically significant at *p* < 0.05. All statistical calculations were performed using the standard software package SPSS v 20.0.

## 5. Conclusions

Our study provides data that are supported with the associated increased expression of CD68+ with the expression of the spike protein within placental tissue, suggesting that inflammatory changes in the placenta favor direct virus transmission.

Also, the finding of a positive correlation between maternal serum levels of TBARS and the expression of spike protein within the placenta, as well as the association between the expression of CD68+ macrophages in placental tissue and TBARS in both maternal and fetal serum, highlights the role of TBARS as an essential biomarker of oxidative stress. TBARS, as a biomarker of oxidative stress and an index of lipid peroxidation, is vital in identifying patients with possible direct viral transmission to the fetus and a high risk of complications in pregnancy and the neonatal period due to oxidative stress and inflammation.

This study has confirmed that Spike protein expression in placental tissue is associated with the newborn’s stay in intensive neonatal care. Therefore, immunoreactivity analysis for the spike antigen is important in detecting newborns at risk of early neonatal complications.

## Figures and Tables

**Table 1:** Mean immunopositivity for spike, ACE2, and CD68+ within the placental tissue of patients infected with SARS-CoV-2 during pregnancy and the control group expressed as mean ± SD. Statistical analysis of the difference in immunopositivity for spike, ACE2, and CD68+ within the placental tissue of patients infected with SARS-CoV-2 during pregnancy and the control groups was performed by the Mann–Whitney test.

Variable	SARS-CoV-2+ Group		Control Group				
	Min–Max	Means ± SD	Min–Max	Means	Test Statistics	Freedom Degree	Significance
SPIKE	0–9	4.44 ± 2.98	0–4	0.73 ± 1.03	4463	-	0.000
ACE2	0–9	3.15 ± 3.13	0–6	1.82 ± 2.28	1605	-	0.108
CD68+	0–39	18.11 ± 8.36	0–16	7.05 ± 4.39	4740	-	0.000

**Table 2:** Analysis of the expression of ACE2, spike proteins, and CD68+ macrophages within the placental tissue of patients infected with the SARS-CoV-2 virus during pregnancy compared with the trimester in which the infection occurred.

	Test Statistics	Freedom Degree	Significance
SPIKE	1579	2	0.454
ACE2	3696	2	0.158
CD68+	0.987	2	0.610

**Table 3:** Analysis of ACE2, spike protein, and CD68+ macrophage expression within the placental tissue of patients infected with the SARS-CoV-2 virus during pregnancy, concerning the severity of maternal clinical presentation.

	Test Statistics	Freedom Degree	Significance
SPIKE	0.180	1	0.857
ACE2	1211	1	0.226
CD68+	0.349	1	0.727

**Table 4:** Statistical analysis of the association of perinatal outcome with the moment of pregnancy when SARS-CoV-2 infection occurred, as tested by Spearman’s rank correlation test.

	Correlation Coefficient	Number of Cases	Significance
Mother’s age	0.136	28	0.482
APGAR Score/1 min	−0.199	28	0.301
APGAR Score/5 min	−0.092	28	0.636
Birth weight	0.017	28	0.929
Birth length	0.142	28	0.462
Head circumference	0.102	28	0.600
gw	−0.042	28	0.828
Placental weight	−0.439	28	0.017
The relation between placental weight and birth Weight	0.396	28	0.033
Umbilical cord	0.056	28	0.772
Length of hospitalization of newborn	0.207	28	0.291
NICU stay length	0.306	28	0.113
IgM level	−0.028	28	0.886

**Table 5:** Statistical analysis of the association of biomarkers of oxidative stress and antioxidant protection (M-mother blood sample; F-fetus blood sample) with the expression of spike proteins within placental tissue by Spearman’s rank correlation test for the significance level of *r* < 0.05.

Parameter	Number of Samples	Level of Significance	Correlation Coefficient
O_2_^−^-M	46	0.676	−0.063
O_2_^−^-N	46	0.515	0.098
H_2_O_2_-M	46	0.121	0.232
H_2_O_2_-N	46	0.234	0.179
NO-M	46	0.392	−0.129
NO-N	46	0.886	0.022
TBARS-M	46	0.048	0.293
TBARS-N	46	0.110	0.293
GSH-M	46	0.762	−0.046
GSH-N	46	0.901	0.019
SOD-M	46	0.322	−0.149
SOD-N	46	0.685	0.061
CAT-M	46	0.056	−0.283
CAT-N	46	0.203	−0.191

O_2_**^−^**-M Superoxide mother, O_2_**^−^**-N Superoxide newborn, H_2_O_2_-M Hydrogen peroxide mother, H_2_O_2_-N Hydrogen peroxide newborn, NO-M Nitric oxide mother, NO-N Nitric oxide newborn, TBARS-M Thiobarbituric Acid Reactive Substances mother, TBARS-N Thiobarbituric Reactive Substances newborn, GSH-M Glutathione mother, GSH-N Glutathione newborn, SOD-M Superoxide dismutase mother, SOD-N Superoxide dismutase newborn, CAT-M Catalase mother, CAT-N Catalase newborn.

**Table 6:** Clinical scoring system for ACE2 and anti-CD68+ antibodies and spike protein.

ACE_2_	Spike Protein	Anti-CD68+
Coloring Intensity: A score of 0–3 is assigned	Positive Cell Percentage Score: Assigned a score of 0–3.	Scored 0–3 according to the number of cells
0: no counting, identical to negative control	0: 0% of stained cells	0: 0 CD68+ cells per HPF
1: poor counting	1: <10% stained cells	1: <10 CD68+ cells per HPF
2: moderate counting	2: 10–50% stained cells	2: 11–50 cells per HPF
3: strong counting	3: >50% stained cells	3: >50 cells per HPF

## Data Availability

All data are available upon request from the corresponding author.

## Abbreviations

COVID-19	Coronavirus disease 2019
NICU	Neonatal Intensive Care Unit
SARS-CoV-2	Severe Acute Respiratory Syndrome Coronavirus 2
FVM	Fetal Vascular Malperfusion
MVM	Maternal Vascular Malperfusion
O_2_^−^	Superoxide
H_2_O_2_	Hydrogen peroxide
NO	Nitric oxide
CAT	Catalase
GPX	Glutathione peroxidase
SOD	Superoxide dismutase
TBARS	Thiobarbituric Acid Reactive Substances
GSH	Glutathione
RT-PCR	Reverse Transcription Polymerase Chain Reaction
ACE2	Angiotensin-converting enzyme 2
TMPRSS2	Type 2 Transmembrane Serine Protease
CD68	Cluster of Differentiation 68 macrophages
gw	Gestational week

## References

[1] Macáková K., Pšenková P., Šupčíková N., Vlková B., Celec P., Záhumenský J. (2023). Effect of SARS-CoV-2 Infection and COVID-19 Vaccination on Oxidative Status of Human Placenta: A Preliminary Study. Antioxidants.

[2] China CDC Weekly (2020). Protocol for Prevention and Control of COVID-19 (Edition 6). China CDC Wkly.

[3] Asselah T., Durantel D., Pasmant E., Lau G., Schinazi R.F. (2021). COVID-19: Discovery, Diagnostics and Drug Development. J. Hepatol..

[4] Zang Y., Gu L., Zhang Y., Wang Y., Xue F. (2018). Identification of Key Genes and Pathways in Uterine Leiomyosarcoma through Bioinformatics Analysis. Oncol. Lett..

[5] Bicanin Ilic M., Nikolic Turnic T., Ilic I., Nikolov A., Mujkovic S., Rakic D., Jovic N., Arsenijevic N., Mitrovic S., Spasojevic M. (2025). SARS-CoV-2 Infection and Its Association with Maternal and Fetal Redox Status and Outcomes: A Prospective Clinical Study. J. Clin. Med..

[6] Andersen K.G., Rambaut A., Lipkin W.I., Holmes E.C., Garry R.F. (2020). The Proximal Origin of SARS-CoV-2. Nat. Med..

[7] Hoffmann M., Kleine-Weber H., Schroeder S., Krüger N., Herrler T., Erichsen S., Schiergens T.S., Herrler G., Wu N.-H., Nitsche A. (2020). SARS-CoV-2 Cell Entry Depends on ACE2 and TMPRSS2 and Is Blocked by a Clinically Proven Protease Inhibitor. Cell.

[8] Wrapp D., Wang N., Corbett K.S., Goldsmith J.A., Hsieh C.-L., Abiona O., Graham B.S., McLellan J.S. (2020). Cryo-EM Structure of the 2019-NCoV Spike in the Prefusion Conformation. Science.

[9] Yan R., Zhang Y., Li Y., Xia L., Guo Y., Zhou Q. (2020). Structural Basis for the Recognition of SARS-CoV-2 by Full-Length Human ACE2. Science.

[10] D’Souza R., Malhamé I., Teshler L., Acharya G., Hunt B.J., McLintock C. (2020). A Critical Review of the Pathophysiology of Thrombotic Complications and Clinical Practice Recommendations for Thromboprophylaxis in Pregnant Patients with COVID-19. Acta Obs. Gynecol. Scand..

[11] Sardu C., Gambardella J., Morelli M.B., Wang X., Marfella R., Santulli G. (2020). Hypertension, Thrombosis, Kidney Failure, and Diabetes: Is COVID-19 an Endothelial Disease? A Comprehensive Evaluation of Clinical and Basic Evidence. J. Clin. Med..

[12] Zhao Y., Zhao Z., Wang Y., Zhou Y., Ma Y., Zuo W. (2020). Single-Cell RNA Expression Profiling of ACE2, the Putative Receptor of Wuhan 2019-NCov. bioRxiv.

[13] Zhu Y., Sharma L., Chang D. (2023). Pathophysiology and Clinical Management of Coronavirus Disease (COVID-19): A Mini-Review. Front. Immunol..

[14] Zhao X., Jiang Y., Zhao Y., Xi H., Liu C., Qu F., Feng X. (2020). Analysis of the Susceptibility to COVID-19 in Pregnancy and Recommendations on Potential Drug Screening. Eur. J. Clin. Microbiol. Infect. Dis..

[15] Levy A., Yagil Y., Bursztyn M., Barkalifa R., Scharf S., Yagil C. (2008). ACE2 Expression and Activity Are Enhanced during Pregnancy. Am. J. Physiol. Regul. Integr. Comp. Physiol..

[16] Joyner J., Neves L.A.A., Granger J.P., Alexander B.T., Merrill D.C., Chappell M.C., Ferrario C.M., Davis W.P., Brosnihan K.B. (2007). Temporal-Spatial Expression of ANG-(1-7) and Angiotensin-Converting Enzyme 2 in the Kidney of Normal and Hypertensive Pregnant Rats. Am. J. Physiol. Regul. Integr. Comp. Physiol..

[17] Brosnihan K.B., Neves L.A.A., Anton L., Joyner J., Valdes G., Merrill D.C. (2004). Enhanced Expression of Ang-(1-7) during Pregnancy. Braz. J. Med. Biol. Res..

[18] Suhren J.-T., Meinardus A., Hussein K., Schaumann N. (2022). Meta-Analysis on COVID-19-Pregnancy-Related Placental Pathologies Shows No Specific Pattern. Placenta.

[19] Levitan D., London V., McLaren R.A., Mann J.D., Cheng K., Silver M., Balhotra K.S., McCalla S., Loukeris K. (2021). Histologic and Immunohistochemical Evaluation of 65 Placentas From Women With Polymerase Chain Reaction–Proven Severe Acute Respiratory Syndrome Coronavirus 2 (SARS-CoV-2) Infection. Arch. Pathol. Lab. Med..

[20] Al-Rawaf S.A., Mousa E.T., Kareem N.M. (2022). Correlation between Pregnancy Outcome and Placental Pathology in COVID-19 Pregnant Women. Infect. Dis. Obs. Gynecol..

[21] Nascimento A.C.M., Avvad-Portari E., Meuser-Batista M., Conde T.C., de Sá R.A.M., Salomao N., Rabelo K., Ciasca E.S., de Oliveira Brendolin M., Vasconcelos Z. (2024). Histopathological and Clinical Analysis of COVID-19-Infected Placentas. Surg. Exp. Pathol..

[22] Baergen R.N., Heller D.S. (2020). Placental Pathology in Covid-19 Positive Mothers: Preliminary Findings. Pediatr. Dev. Pathol..

[23] Hosier H., Farhadian S.F., Morotti R.A., Deshmukh U., Lu-Culligan A., Campbell K.H., Yasumoto Y., Vogels C.B.F., Casanovas-Massana A., Vijayakumar P. (2020). SARS–CoV-2 Infection of the Placenta. J. Clin. Investig..

[24] Smithgall M.C., Liu-Jarin X., Hamele-Bena D., Cimic A., Mourad M., Debelenko L., Chen X. (2020). Third-trimester Placentas of Severe Acute Respiratory Syndrome Coronavirus 2 (SARS-CoV-2)-positive Women: Histomorphology, Including Viral Immunohistochemistry and *In-situ* Hybridization. Histopathology.

[25] Shanes E.D., Mithal L.B., Otero S., Azad H.A., Miller E.S., Goldstein J.A. (2020). Placental Pathology in COVID-19. Am. J. Clin. Pathol..

[26] Prabhu M., Cagino K., Matthews K., Friedlander R., Glynn S., Kubiak J., Yang Y., Zhao Z., Baergen R., DiPace J. (2020). Pregnancy and Postpartum Outcomes in a Universally Tested Population for SARS-CoV-2 in New York City: A Prospective Cohort Study. BJOG.

[27] Glynn S.M., Yang Y.J., Thomas C., Friedlander R.L., Cagino K.A., Matthews K.C., Riley L.E., Baergen R.N., Prabhu M. (2022). SARS-CoV-2 and Placental Pathology. Am. J. Surg. Pathol..

[28] Mulvey J.J., Magro C.M., Ma L.X., Nuovo G.J., Baergen R.N. (2020). Analysis of Complement Deposition and Viral RNA in Placentas of COVID-19 Patients. Ann. Diagn. Pathol..

[29] Patberg E.T., Adams T., Rekawek P., Vahanian S.A., Akerman M., Hernandez A., Rapkiewicz A.V., Ragolia L., Sicuranza G., Chavez M.R. (2021). Coronavirus Disease 2019 Infection and Placental Histopathology in Women Delivering at Term. Am. J. Obstet. Gynecol..

[30] Hsu A.L., Guan M., Johannesen E., Stephens A.J., Khaleel N., Kagan N., Tuhlei B.C., Wan X. (2021). Placental SARS-CoV-2 in a Pregnant Woman with Mild COVID-19 Disease. J. Med. Virol..

[31] Shynlova O., Nadeem L., Zhang J., Dunk C., Lye S. (2020). Myometrial Activation: Novel Concepts Underlying Labor. Placenta.

[32] Hamilton S., Oomomian Y., Stephen G., Shynlova O., Tower C.L., Garrod A., Lye S.J., Jones R.L. (2012). Macrophages Infiltrate the Human and Rat Decidua During Term and Preterm Labor: Evidence That Decidual Inflammation Precedes Labor1. Biol. Reprod..

[33] Azinheira Nobrega Cruz N., Stoll D., Casarini D.E., Bertagnolli M. (2021). Role of ACE2 in Pregnancy and Potential Implications for COVID-19 Susceptibility. Clin. Sci..

[34] Lye P., Dunk C.E., Zhang J., Wei Y., Nakpu J., Hamada H., Imperio G.E., Bloise E., Matthews S.G., Lye S.J. (2021). ACE2 Is Expressed in Immune Cells That Infiltrate the Placenta in Infection-Associated Preterm Birth. Cells.

[35] Krop J., van der Meeren L.E., van der Hoorn M.L.P., Ijsselsteijn M.E., Dijkstra K.L., Kapsenberg H., van der Keur C., Cornish E.F., Nikkels P.G.J., Koning F. (2023). Identification of a Unique Intervillous Cellular Signature in Chronic Histiocytic Intervillositis. Placenta.

[36] Joerink M., Rindsjö E., Van Riel B., Alm J., Papadogiannakis N. (2011). Placental Macrophage (Hofbauer Cell) Polarization Is Independent of Maternal Allergen-Sensitization and Presence of Chorioamnionitis. Placenta.

[37] Resta L., Vimercati A., Cazzato G., Fanelli M., Scarcella S.V., Ingravallo G., Colagrande A., Sablone S., Stolfa M., Arezzo F. (2022). SARS-CoV-2, Placental Histopathology, Gravity of Infection and Immunopathology: Is There an Association?. Viruses.

[38] Traeder J., Jonigk D., Feist H., Bröcker V., Länger F., Kreipe H., Hussein K. (2010). Pathological Characteristics of a Series of Rare Chronic Histiocytic Intervillositis of the Placenta. Placenta.

[39] Bos M., Nikkels P.G.J., Cohen D., Schoones J.W., Bloemenkamp K.W.M., Bruijn J.A., Baelde H.J., van der Hoorn M.L.P., Turner R.J. (2018). Towards Standardized Criteria for Diagnosing Chronic Intervillositis of Unknown Etiology: A Systematic Review. Placenta.

[40] Sato Y., Maekawa K., Aman M., Yamashita A., Kodama Y., Maki Y., Sameshima H., Asada Y. (2019). CD39 Downregulation in Chronic Intervillositis of Unknown Etiology. Virchows Arch..

[41] Capuani C., Meggetto F., Duga I., Danjoux M., March M., Parant O., Brousset P., Aziza J. (2013). Specific Infiltration Pattern of FOXP3+ Regulatory T Cells in Chronic Histiocytic Intervillositis of Unknown Etiology. Placenta.

[42] Becker J., Qiu D., Baron W., Wilting J. (2023). Immunofluorescence Studies on the Expression of the SARS-CoV-2 Receptors in Human Term Placenta. Cells Tissues Organs.

[43] Caparros-Gonzalez R.A., Pérez-Morente M.A., Hueso-Montoro C., Álvarez-Serrano M.A., de la Torre-Luque A. (2020). Congenital, Intrapartum and Postnatal Maternal-Fetal-Neonatal SARS-CoV-2 Infections: A Narrative Review. Nutrients.

[44] De Luca D., Vauloup-Fellous C., Benachi A., Vivanti A. (2023). Transmission of SARS-CoV-2 from Mother to Fetus or Neonate: What to Know and What to Do?. Semin. Fetal Neonatal Med..

[45] Gardella B., Dominoni M., Scatigno A.L., Cesari S., Fiandrino G., Orcesi S., Spinillo A. (2022). What Is Known about Neuroplacentology in Fetal Growth Restriction and in Preterm Infants: A Narrative Review of Literature. Front. Endocrinol..

[46] Roescher A.M., Timmer A., Erwich J.J.H.M., Bos A.F. (2014). Placental Pathology, Perinatal Death, Neonatal Outcome, and Neurological Development: A Systematic Review. PLoS ONE.

[47] Benski C., Di Filippo D., Taraschi G., Reich M.R. (2020). Guidelines for Pregnancy Management During the COVID-19 Pandemic: A Public Health Conundrum. Int. J. Envron. Res. Public Health.

[48] Gullo G., Scaglione M., Buzzaccarini G., Laganà A.S., Basile G., Chiantera V., Cucinella G., Zaami S. (2022). Cell-Free Fetal DNA and Non-Invasive Prenatal Diagnosis of Chromosomopathies and Pediatric Monogenic Diseases: A Critical Appraisal and Medicolegal Remarks. J. Pers. Med..

[49] Riemma G., De Franciscis P., Tesorone M., Coppa E., Schiattarella A., Billone V., Lopez A., Cucinella G., Gullo G., Carotenuto R.M. (2023). Obstetric and Gynecological Admissions and Hospitalizations in an Italian Tertiary-Care Hospital during COVID-19 Pandemic: A Retrospective Analysis According to Restrictive Measures. J. Clin. Med..

[50] Tsermpini E.E., Glamočlija U., Ulucan-Karnak F., Redenšek Trampuž S., Dolžan V. (2022). Molecular Mechanisms Related to Responses to Oxidative Stress and Antioxidative Therapies in COVID-19: A Systematic Review. Antioxidants.

[51] Lage S.L., Amaral E.P., Hilligan K.L., Laidlaw E., Rupert A., Namasivayan S., Rocco J., Galindo F., Kellogg A., Kumar P. (2022). Persistent Oxidative Stress and Inflammasome Activation in CD14highCD16− Monocytes From COVID-19 Patients. Front. Immunol..

[52] Imanparast F., Hashemi B., Mokhtari F., Mohaghegh P., Azar F.F., Mehvari F. (2024). The Effect of Mother’s Age on the Neonatal Cord Serum’s Oxidative Stress Index and Maternal and Neonatal Outcomes: A Case Control Study. BMC Pregnancy Childbirth.

[53] Radan A.-P., Baud D., Favre G., Papadia A., Surbek D., Baumann M., Raio L. (2022). Low Placental Weight and Altered Metabolic Scaling after Severe Acute Respiratory Syndrome Coronavirus Type 2 Infection during Pregnancy: A Prospective Multicentric Study. Clin. Microbiol. Infect..

[54] Bloise E., Zhang J., Nakpu J., Hamada H., Dunk C.E., Li S., Imperio G.E., Nadeem L., Kibschull M., Lye P. (2021). Expression of Severe Acute Respiratory Syndrome Coronavirus 2 Cell Entry Genes, Angiotensin-Converting Enzyme 2 and Transmembrane Protease Serine 2, in the Placenta across Gestation and at the Maternal-Fetal Interface in Pregnancies Complicated by Preterm Birth or Preeclampsia. Am. J. Obstet. Gynecol..

[55] Pique-Regi R., Romero R., Tarca A.L., Luca F., Xu Y., Alazizi A., Leng Y., Hsu C.-D., Gomez-Lopez N. (2020). Does the Human Placenta Express the Canonical Cell Entry Mediators for SARS-CoV-2?. Elife.

[56] Pringle K.G., Tadros M.A., Callister R.J., Lumbers E.R. (2011). The Expression and Localization of the Human Placental Prorenin/Renin-Angiotensin System throughout Pregnancy: Roles in Trophoblast Invasion and Angiogenesis?. Placenta.

[57] Valdés G., Neves L.A.A., Anton L., Corthorn J., Chacón C., Germain A.M., Merrill D.C., Ferrario C.M., Sarao R., Penninger J. (2006). Distribution of Angiotensin-(1-7) and ACE2 in Human Placentas of Normal and Pathological Pregnancies. Placenta.

[58] Glowacka I., Bertram S., Herzog P., Pfefferle S., Steffen I., Muench M.O., Simmons G., Hofmann H., Kuri T., Weber F. (2010). Differential Downregulation of ACE2 by the Spike Proteins of Severe Acute Respiratory Syndrome Coronavirus and Human Coronavirus NL63. J. Virol..

[59] Lim M.J., Lakshminrusimha S., Hedriana H., Albertson T. (2023). Pregnancy and Severe ARDS with COVID-19: Epidemiology, Diagnosis, Outcomes and Treatment. Semin. Fetal Neonatal Med..

[60] Merrill D.C., Karoly M., Chen K., Ferrario C.M., Brosnihan K.B. (2002). Angiotensin-(1-7) in Normal and Preeclamptic Pregnancy. Endocrine.

[61] West C.A., Sasser J.M., Baylis C. (2016). The Enigma of Continual Plasma Volume Expansion in Pregnancy: Critical Role of the Renin-Angiotensin-Aldosterone System. Am. J. Physiol. Ren. Physiol..

[62] Ferrario C.M., Trask A.J., Jessup J.A. (2005). Advances in Biochemical and Functional Roles of Angiotensin-Converting Enzyme 2 and Angiotensin-(1–7) in Regulation of Cardiovascular Function. Am. J. Physiol. Heart Circ. Physiol..

[63] Narang K., Enninga E.A.L., Gunaratne M.D.S.K., Ibirogba E.R., Trad A.T.A., Elrefaei A., Theiler R.N., Ruano R., Szymanski L.M., Chakraborty R. (2020). SARS-CoV-2 Infection and COVID-19 During Pregnancy: A Multidisciplinary Review. Mayo Clin. Proc..

[64] Ghadhanfar E., Alsalem A., Al-Kandari S., Naser J., Babiker F., Al-Bader M. (2017). The Role of ACE2, Angiotensin-(1–7) and Mas1 Receptor Axis in Glucocorticoid-Induced Intrauterine Growth Restriction. Reprod. Biol. Endocrinol..

[65] Bharadwaj M.S., Strawn W.B., Groban L., Yamaleyeva L.M., Chappell M.C., Horta C., Atkins K., Firmes L., Gurley S.B., Brosnihan K.B. (2011). Angiotensin-Converting Enzyme 2 Deficiency Is Associated with Impaired Gestational Weight Gain and Fetal Growth Restriction. Hypertension.

[66] Tamanna S., Clifton V.L., Rae K., van Helden D.F., Lumbers E.R., Pringle K.G. (2020). Angiotensin Converting Enzyme 2 (ACE2) in Pregnancy: Preeclampsia and Small for Gestational Age. Front. Physiol..

[67] Maranto M., Zaami S., Restivo V., Termini D., Gangemi A., Tumminello M., Culmone S., Billone V., Cucinella G., Gullo G. (2023). Symptomatic COVID-19 in Pregnancy: Hospital Cohort Data between May 2020 and April 2021, Risk Factors and Medicolegal Implications. Diagnostics.

[68] Hecht J.L., Quade B., Deshpande V., Mino-Kenudson M., Ting D.T., Desai N., Dygulska B., Heyman T., Salafia C., Shen D. (2020). SARS-CoV-2 Can Infect the Placenta and Is Not Associated with Specific Placental Histopathology: A Series of 19 Placentas from COVID-19-Positive Mothers. Mod. Pathol..

[69] Verma S., Joshi C.S., Silverstein R.B., He M., Carter E.B., Mysorekar I.U. (2021). SARS-CoV-2 Colonization of Maternal and Fetal Cells of the Human Placenta Promotes Alteration of Local Renin-Angiotensin System. Med.

[70] Wang C., Zhou Y.-H., Yang H.-X., Poon L.C. (2020). Intrauterine Vertical Transmission of SARS-CoV-2: What We Know so Far. Ultrasound Obs. Gynecol..

[71] Dong L., Tian J., He S., Zhu C., Wang J., Liu C., Yang J. (2020). Possible Vertical Transmission of SARS-CoV-2 From an Infected Mother to Her Newborn. J. Am. Med. Assoc..

[72] World Health Organization (2021). Definition and Categorization of the Timing of Mother-to-Child Transmission of SARS-CoV-2.

[73] Chi H., Chiu N.C., Tai Y.L., Chang H.Y., Lin C.H., Sung Y.H., Tseng C.Y., Liu L.Y.M., Lin C.Y. (2021). Clinical Features of Neonates Born to Mothers with Coronavirus Disease-2019: A Systematic Review of 105 Neonates. J. Microbiol. Immunol. Infect..

[74] Huntley B.J.F., Huntley E.S., Di Mascio D., Chen T., Berghella V., Chauhan S.P. (2020). Rates of Maternal and Perinatal Mortality and Vertical Transmission in Pregnancies Complicated by Severe Acute Respiratory Syndrome Coronavirus 2 (SARS-Co-V-2) Infection. Obstet. Gynecol..

[75] Duran P., Berman S., Niermeyer S., Jaenisch T., Forster T., Gomez Ponce de Leon R., De Mucio B., Serruya S. (2020). COVID-19 and Newborn Health: Systematic Review. Rev. Panam. Salud Pública.

[76] Gullo G., Scaglione M., Cucinella G., Riva A., Coldebella D., Cavaliere A.F., Signore F., Buzzaccarini G., Spagnol G., Laganà A.S. (2022). Congenital Zika Syndrome: Genetic Avenues for Diagnosis and Therapy, Possible Management and Long-Term Outcomes. J. Clin. Med..

[77] Taglauer E., Benarroch Y., Rop K., Barnett E., Sabharwal V., Yarrington C., Wachman E.M. (2020). Consistent Localization of SARS-CoV-2 Spike Glycoprotein and ACE2 over TMPRSS2 Predominance in Placental Villi of 15 COVID-19 Positive Maternal-Fetal Dyads. Placenta.

[78] Edlow A.G., Li J.Z., Collier A.Y., Atyeo C., James K.E., Boatin A.A., Gray K.J., Bordt E.A., Shook L.L., Yonker L.M. (2020). Assessment of Maternal and Neonatal SARS-CoV-2 Viral Load, Transplacental Antibody Transfer, and Placental Pathology in Pregnancies During the COVID-19 Pandemic. J. Am. Med. Assoc. Netw Open.

[79] Fuentes-Zacarías P., Murrieta-Coxca J.M., Gutiérrez-Samudio R.N., Schmidt A., Schmidt A., Markert U.R., Morales-Prieto D.M. (2021). Pregnancy and Pandemics: Interaction of Viral Surface Proteins and Placenta Cells. Biochim. Biophys. Acta Mol. Basis Dis..

[80] Facchetti F., Bugatti M., Drera E., Tripodo C., Sartori E., Cancila V., Papaccio M., Castellani R., Casola S., Boniotti M.B. (2020). SARS-CoV2 Vertical Transmission with Adverse Effects on the Newborn Revealed through Integrated Immunohistochemical, Electron Microscopy and Molecular Analyses of Placenta. EBioMedicine.

[81] Guo X., Semerci N., De Assis V., Kayisli U.A., Schatz F., Steffensen T.S., Guzeloglu-Kayisli O., Lockwood C.J. (2022). Regulation of Proinflammatory Molecules and Tissue Factor by SARS-CoV-2 Spike Protein in Human Placental Cells: Implications for SARS-CoV-2 Pathogenesis in Pregnant Women. Front. Immunol..

[82] Incognito G.G., Distefano R.E.C., Campo G., Gulino F.A., Gulisano C., Gullotta C., Gullo G., Cucinella G., Tuscano A., Bruno M.T. (2023). Comparison of Maternal and Neonatal Outcomes between SARS-CoV-2 Variants: A Retrospective, Monocentric Study. J. Clin. Med..

[83] Garg R., Agarwal R., Yadav D., Singh S., Kumar H., Bhardwaj R. (2023). Histopathological Changes in Placenta of Severe Acute Respiratory Syndrome Coronavirus 2 (SARS-Cov-2) Infection and Maternal and Perinatal Outcome in COVID-19. J. Obstet. Gynecol. India.

[84] Maranto M., Gullo G., Bruno A., Minutolo G., Cucinella G., Maiorana A., Casuccio A., Restivo V. (2023). Factors Associated with Anti-SARS-CoV-2 Vaccine Acceptance among Pregnant Women: Data from Outpatient Women Experiencing High-Risk Pregnancy. Vaccines.

[85] Kim S.-W., Roh J., Park C.-S. (2016). Immunohistochemistry for Pathologists: Protocols, Pitfalls, and Tips. J. Pathol. Transl. Med..

[86] Fischer A.H., Jacobson K.A., Rose J., Zeller R. (2008). Hematoxylin and Eosin Staining of Tissue and Cell Sections. Cold Spring Harb. Protoc..

